# Halitosis in Otorhinolaryngology Practice

**Published:** 2015-03

**Authors:** Ozan Gokdogan, Tolgahan Catli, Fikret Ileri

**Affiliations:** 1 *Department of Otorhinolaryngology, Ankara Memorial Hospital, Ankara, Turkey.*; 2 *Department of Otorhinolaryngology, Bozyaka Teaching and Research Hospital, Izmir,Turkey.*; 3 **Department of Otorhinolaryngology, School of Medicine, Gazi University, Ankara, Turkey.**

**Keywords:** Halitosis, Halitophobia, Halitophobia, Oral pathology, Pseudohalitosis, Volatile sulphur compounds

## Abstract

Halitosis is a common and devastating condition, which may affect up to 1/3 of the population. It can be classified either as genuine halitosis, pseudohalitosis, or halitophobia. Genuine halitosis is more common and usually related to an organic pathology such as periodontitis. Malodour molecules such as sulfur compounds that arise from bacterial interactions generate the basis of oral malodour. Pathologies of the tongue, poor oral hygiene, deep caries, cryptic tonsillary hypertrophia, and postnasal drainage are also associated with halitosis. Gastro-esophageal pathologies and systemic problems are accepted as extra-oral sources of halitosis. There are various methods for the diagnosis of halitosis including objective and subjective methods. General oral hygiene recommendations and specific interventions for the related etiological factors have to be addressed in order to achieve satisfactory results after the treatment. Clinicians have to be aware of these aspects regarding this unfavorable condition to achieve the best results.

## Introduction

There are plenty of existing reports, regarding the etiology and management of halitosis and its effects on the quality of life of patients (1,2). Although it is generally accepted as a subjective symptom; in some cases, it can easily and seriously affect patients’ communication. However, diagnosis is also complicated because some of the patients are not able to recognize their own symptoms. Though objective methods in the diagnosis of halitosis have been developing; unfortunately they are infrequent and limited in numbers. There is no existing agreement on the epidemiology of halitosis. In many articles, the incidence varies from 2% to 27.5%. There was no relationship between “age, sex” and “halitosis” in studies (3,4). 

Transient malodour of the breath may be evident in the early times of the morning and may have alternations during the daytime. Oral diet may also affect the odourity of the breath. 

Tobacco and alcohol consumption or food intake like garlic or some spicy foods can cause a transient odour change in breath (1). Xserostomia, as a result of various diseases, or as a result of medications like antidepressants, antipsycotics, and narcotics may also cause or exacerbate halitosis. Some medications such as cyclosporine and fish oil derivative, which is used in the treatment of Crohn’s disease, may also contribute to halitosis (5-7).

## Etiology

Halitosis can be described as a sensation of unpleasant or bad smelling breath. Bad or foul breath, breath malodour, oral malodour, foetor ex-ore, and foetor oris are all terms used instead of halitosis. It is not a rare problem, it exists all over the world, and may affect personal life because it influences personal communication. Generally halitosis can be classified into three groups: genuine halitosis, pseudohalitosis, and halitophobia (Table.1). 

**Table 1:** Halitosis classifications

**Genuine Halitosis**	**Pseudohalitosis**	**Halitophobia**
**a.** Physiologic al halitosis **b.** Pathological halitosis -Oral -Extra-oral	Halitosis is not perceived by others. Condition improves with counseling and simple measures	No obvious signs of halitosis but patient persists in having halitosis (generally after the adequate treatment of genuine or pseudohalitosis)

Genuine halitosis is the classification of obvious malodour that goes beyond socially acceptable levels and can be classified as physiologic or pathologic halitosis. Non-genuine halitosis is diagnosed approximately in 27% of all patients complaining from halitosis (8). Pseudo-halitosis can be described as a situation where obvious malodour is not perceived by others and only perceived by the patient. Halitophobia is a situation when a patient complains about halitosis after the treatment of either genuine halitosis or pseudo-halitosis even though no objective clues can be determined during physical examination. Patients with halitophobia may require neurologic and psychiatric evaluation (9). 

## Clinical Presentation

Halitosis is caused by oral problems in 80-90% of cases (10). Chronic pathologies, such as periodontal disease, and chronic action of bacteria coating the tongue are the main causes of halitosis. Peri-implant disease, deep carious lesions, exposed necrotic tooth pulps, pericoronitis, mucosal ulcerations, healing (mucosal) wounds, impacted food or debris, imperfect dental restorations, unclean dentures, and factors causing decreased salivary flow rate can also be the cause (11,12). Chronic sinusitis, post-nasal drip and nasal foreign bodies, respiratory tract infections and malign diseases, gastrointestinal problems such as reflux, inflammatorry bowel disease, helicobacter pylori infection, and Zenker’s diverticle are the non-oral factors which can result in halitosis (13-15). Stressful factors may have a predisposing effect on halitosis (16,17). Parasitosis is suggested as a possible etiological factor in children (18). The odour of trimethylaminuria in the breath can cause strong halitosis which is called ‘fish odour syndrome’; whereas diabetic ketoacidosis, renal failure, and hepatic failure can also cause specific odours in the breath (19). Oral malodour is usually caused by microbial degradation of organic substrates such as glucose, mucins, and especially proteins (20,21). Broken down components of ephitelial cells, salivary and serum proteins, and food debris interact with bacteria and turn into malodour molecules (22). Malodour molecules are produced by putrefaction of debris and protein substrates accompagnied by the proteolytic effect of bacterial species. Various species such as “Actinobacillus actinomycetemcomitans, actinomyces species, Atopbium parvulum, Camplyobacter rectus, Desulfovibro species, Eikenella corrodens, Eubacterium sulci, Fusobacterium nucleatum, Peptostrepto- coccus micros, Porphyromonas endodontalis, Porphyromonas gingivalis, Solobacterium moorei, Bacteriodes forsythus, Treponema denticola, (and) Prevotella intermedia” are closely related with the putrefaction process (20,23-25).

The common source of proteins causing malodour are salivary mucins and ephitelial cell components which contain various glycoproteins (26). Glycoprotein lysis starts with the detachement of the protein from the carbohydrate part. This step is necessary for further protein processing steps and usually results from gram positive bacterial activity (27). Streptococcus salivarious, which is the main bacteria of saliva, promotes the mucin putrefaction effect of Porphyromonas gingivalis (28). Proteolysis and amino acid utilisation of the protein core are generally made by gram negative bacteria (29). These compounds consist of volatile sulphur compounds (VSCs) and some other products (30). The sulphur compounds are usually generated from the proteolysis of sulphur-containing amino acids such as cysteine, cystine, and methionine as well as tryptophan and lysine (1,31). Volatile sulphur compounds such as methyl mercaptan (MM), hydrogen sulphide (HS), and dimethyl sulphide (DMS), in addition to some other compounds contribute to malodour (1,19). Furthermore, sulphur compounds, short-chain fatty acids (butyrate, propionate, valerate), diamines (cadaverine, putrescine), alcohols, phenyl compounds (indole, skatole, phyridiene), alkines, ketones, and nitrogen-containing compounds (urea, ammonia) may contribute to the process (30,32). The concentration of odorant molecules in halitotic breath must exceed a treshold in order to be detected by either subjective or objective methods. The etiological organisms are mostly related with chronic periodontitis and the products are most commonly found in both adjacent gingival tissue and saliva. The dorsum of the tongue was the major site for VSCs, after periodontal tissue, as well as every cript of the oral cavity such as the tongue papilla, tonsillary cript, etc. (1,31). These potential spaces filled with desquamated ephitelial cells, food debris, bacteria, and saliva result in the generation of malodour compounds. Biofilm formation also occurs in such places. Gram positive bacteria is generally found in the outer layer of biofilms and has β-galactosidase activity. Gram negative bacterias are found in deeper layers and produce VSCs with BANA (benzoyl-DL-arginine-naphtylamide) (29). Total gram positive and gram negative anaerobic bacterial load and the β-galactosidase activity of Streptococcus species are important in the generation of oral malodour (33). In contrast some bacterial species, which can be considered as good bacterias, because they are predominantly found in a healty mouth, are noticeably absent in patients suffering from oral malodour (34).

Extra-oral halitosis has origins outside the mouth. Extra-oral halitosis can be divided into two groups: blood-borne and non-blood-borne halitosis (35). The majority of extra-oral halitosis are blood-borne. In extra-oral blood borne halitosis, volatile odour compounds (VOCs) generate from any part of the body, travel to the lungs through the vascular system, and enter the alveoli through gas exchange. VOCs get out of the body with exhalation. Systemic diseases including hepatic failure/liver cirrhosis, uremia/kidney failure, diabetic ketoacidosis, diabetes mellitus, metabolic disorders including isolated persistent hypermethio- ninemia and fish odor syndrome, food intake like garlic or onion, and medications like disulfiram, dimethyl sulfoxide, cysteamine have been reported as the etiology of halitosis in literature (35-40). Blood borne halitosis is also frequently caused by VOCs especially by DMS which originates from the gut (35,36). With the increased porto caval shunts DMS concentration increases in the blood. DMS diffuses into the alveoli through the vascular system in the lungs (38). VOC measurement provides information about the underlying pathology. Altough blood measures of VOCs may also give information and generally correlate with the air levels, VOC measurements from the breath is preferred for diagnosis^41^. Early after garlic or onion intake, the thiol allyl mercaptan, which contains a reactive –SH group is detected only from the mouth and not from alveolar air. Allyl mercaptan is not stable in the blood so it cannot travel throuh the blood without interaction (35,36,39,42). After 3 hours, neutral sulfide allyl methyl sulfide (AMS), which originates in the gut, was the predominant gas found in both mouth and exhaled breath. This causes the long duration of halitosis after garlic or onion intake (42). In extra-oral non-blood-borne halitosis, the causative odours were not yet identified exactly. Nasal infections are the most important cause of this situation (43). Gastrointestinal problems are also accused as the generator of oral malodour. It was shown that Helicobacter Pylori (HP) was able to produce the VSCs of HS and MM, which suggests that HP may contribute to the development of halitosis (44). The way that VSCs formed by HP in the stomach are transported through the stomach wall into the blood and diffuse into the lung. But there is still debate about its effect on halitosis (45).

## Diagnosis

Altough oral malodour is subjective in nature, some objective tests have been used for clinical assessment. Self-assessment of oral malodour is important and must be considered as the main problem of the patient in social life. However, it is not so reliable, especially in research (46). In organoleptic assessment of oral malodour trained and calibrated professionals assess patients’ breath through a plastic tube, which is inserted into the patients’ mouth in order to prevent the dilution of the mouth with room air. Generally the patient and examiner are separated with a screen wich has a hole for a straw or a tube. This method is relatively subjective and can be affected by patients’ diet, even though it is very easy to perform. An organoleptic measurement method, called “spoon test”, is also very easy to use. A spoon or similar instrument is placed on the dorsum of the tongue and scraped material is smelled (43). Some other objective methods such as VSC measurements with electrochemical reactions like halimeter, gas chromoto- graphy or salivary investigation for bacterial load and other compounds are used in the objective assessment of oral malodour. Halimeter is a portable VSC detector and widely used due to its easy operation. This test only detects VSC products; whereas some other compounds such as volatile short-chain fatty acids, polyamines, alcohols, phenyl compounds, alkanes, ketones, and nitrogen-containing compounds may also contribute to oral malodour (9,47,48). Gas chromotography is believed to give the most objective result such as concentration of specific VSCs in samples of saliva, tongue coating or exhaled breath in oral malodour (59). Samples are analyzed with a flame photometric detector and produce a mass spectra (50). The etiology of oral malodour can be specifically identified with this test; therefore this test is the main objective diagnostic method in research (51). β-galactosidase activity measures the deglycosylation step of glycoprotein lysis. Using a chromogenic substrate that is absorbed onto a chromotography paper disc, β-galactosidase activity can be quantified. Saliva is applied on this paper and a colour change is observed. The β-galactosidase activity assay scores are significantly associated with organoleptic scores (27,29). A salivary incubation test is done by collecting saliva in a glass, wich is put under specific conditions (37°C and in aerobic chamber). After several hours odour can be measured by the examiner. The salivary incubation test is correlated with other tests and much less affected from patients’ dietary rather than other diagnostic tests. An ammonia test was performed by rinsing the mouth at least 30 seconds with urea solution followed by closing the mouth for approximately 5 minutes. With a detector the concentration of ammonia produced by bacteria can be read from a scale. Generally ammonia levels correlate with VSC levels (32). The ninhydrin method is used for detecting low-molecular-weight amines contained in breath. This test is rapid, easy, and correlates well with other tests (52). Real-time polymerase chain reaction (PCR) using the TaqMan system can also be used for detecting VSC producing bacterias. This amplification method allows rapid detection and quantification of oral bacterial DNA (23,53). 

## Management

The first line of management should be targeted to the identified causal factors. In the case of periodontal diseases, appropriate periodontal management must be the first step after examination (54). The benefical effect of oral antiseptic agents - such as clorhexidine and formulations of clorhexidine with cetylpyridium chloride and zinc ions with a combination of periodontal therapy - have been especially shown in some studies (55,56). The patients are recommended to use oral hygiene procedures including tooth cleaning (brushing and interdental flossing) and tongue cleaning; in addition to antimicrobial toothpastes or mouth washes. Cells, food particles, microorganisms and their products must be removed by gentle and regular tongue cleaning. Altough tongue cleaning with brushes or scrapers is recommended in guidelines, the efficacy of this approach is not well demonstrated (57-59). Also the effect of tongue scraping is temporary; and only has a duration of 30 minutes (58). Toothpastes have variable; but generally short time effects as well. Mouthwashes are also widely used. Daily use, two or three times a day for at least 30 seconds, is generally recommended. Zinc and copper are important minerals for olfactory and taste function; as well as vitamin A and vitamin B12. Their defficiency may contribute to or aggravate complaints regarding halitosis. Zinc and copper ions are also used in the treatment. They have the effect of directly neutralizing VSCs; in addition to beneficial effects like antibacterial action. Tooth pastes and mouth washes, especially containing zinc ions, can also neutralize VSCs. Alcohol, phemol, and clorhexidine, which are included in mouth washes, may also mask oral malodour (52,60,61). Commonly used antibiotics may decrease bacterial load and may mask oral malodour. However, the use of antibiotics can also remove all normal flora bacterias. Use of probiotics can also be recommended not only for halitosis but also for preventing dental caries (26,62).

Streptococcus salivarious is the major colonizer of bacteria of the oral cavity and is known to produce bactriocins and bacteriocin-like inhibitory substances. This feature makes this bacterial species good candidates for the development of oral probiotics, especially against oral infectious diseases. BLIS K12 Throat Guard lozenges (BLIS Technologies, Centre for Innovation, Dunedin, New Zealand) contain the original form of Streptococcus salivarious and show antibacterial activity in the oral cavity (63). The bacteria produce antibacterial peptides such as Salivaricin A and Salivaricin B, which are bacteriocins (64,65). In studies, these species of bacteria have an antagonist activity on Streptococcus anginosis T29, Eubacterium saburreum, and Micromonas micros, which have a role on the development of oral malodour as well as pharyngitis in children (63, 66). Therefore, probiotics are helpful and safe treatment alternatives against several bacteria, which have role in the development of halitosis. In non-genuine halitosis, before associating halitosis to a psychiatric problem, organic reasons must be excluded. Especially in non-genuine halitosis the patient must be evaluated for depression and psychogenic disorders. “Olfactory reference syndrome” is one of the psychiatric syndromes, which is characterized by the sensation of bad odors, including halitosis, which are emanating from their body and perceived by others, even from far away (67). For long term effect, the main causes of the disease must be treated. The etiology and management of halitosis must be described to the patient in details. Systemic problems must always be kept in mind and further examination for these causes must be performed if any suspicions are present. Habits like smoking and intaking foods like onion, garlic, and others must be avoided. Eating regular meals and finishing meals with vegetables and fruits, such as carrots or pineapples, must also be recommended to the patient. Recommendations for obtaining good oral hygiene must be the basis of treatment.

## Conclusion

Halitosis is not an uncommon health problem, which affects over 1/3 of the population. Although oral pathologies are the main causative factors, extra-oral pathologies can also be the source, and these must be managed carefully. It must be kept in mind that halitosis might be a manifestation of serious health problems such as cancer. The main diagnostic methods are organoleptic assessment, halimeter, and gas chromotography. All these tests have advantages and restrictions and necessary tests must be chosen according to some variable factors like patient properties, equipment availability and research characteristics. Otorhinolaryn- gologist, periodontologist or gastroentero- logist observations need to be planned in order to detect other causative factors. Generally patients with depressive traits in both groups of genuine haltosis or non genuine halitosis are referred to a psychiatric evaluation. 
